# Factors Associated with Gonioscopy Before Glaucoma Procedures in the IRIS^®^ Registry

**DOI:** 10.21203/rs.3.rs-5789587/v1

**Published:** 2025-01-13

**Authors:** Daniel M. Vu, Joshua B. Gilbert, Eric A. Goldberg, Adam L. Rothman, Michael M. Lin, Ta C. Chang, Sarah H. Van Tassel, Nimesh A. Patel, Nazlee Zebardast, Connor J. Ross, Tobias Elze, Alice C. Lorch, Joan W. Miller

**Affiliations:** Department of Ophthalmology, Massachusetts Eye and Ear, Harvard Medical School, Boston, MA; Department of Ophthalmology, Massachusetts Eye and Ear, Harvard Medical School, Boston, MA; Department of Ophthalmology, Massachusetts Eye and Ear, Harvard Medical School, Boston, MA; Department of Ophthalmology, Bascom Palmer Eye Institute, University of Miami School of Medicine, Miami, FL; Department of Ophthalmology, Massachusetts Eye and Ear, Harvard Medical School, Boston, MA; Department of Ophthalmology, Bascom Palmer Eye Institute, University of Miami School of Medicine, Miami, FL; Israel Englander Department of Ophthalmology, Weill Cornell Medicine, New York, NY; Department of Ophthalmology, Massachusetts Eye and Ear, Harvard Medical School, Boston, MA; Department of Ophthalmology, Massachusetts Eye and Ear, Harvard Medical School, Boston, MA; Department of Ophthalmology, Massachusetts Eye and Ear, Harvard Medical School, Boston, MA; Department of Ophthalmology, Massachusetts Eye and Ear, Harvard Medical School, Boston, MA; Department of Ophthalmology, Massachusetts Eye and Ear, Harvard Medical School, Boston, MA; Department of Ophthalmology, Massachusetts Eye and Ear, Harvard Medical School, Boston, MA

**Keywords:** Gonioscopy, Practice patterns, IRIS Registry

## Abstract

**Purpose:**

To analyze nationwide pre-operative gonioscopy utilization patterns for various glaucoma surgeries and laser surgeries over time using the IRIS^®^ Registry (Intelligent Research in Sight).

**Design:**

Retrospective cohort study.

**Participants:**

All adults who underwent a glaucoma surgery or laser surgery between January 1, 2014 and April 14, 2023.

**Methods:**

The first glaucoma procedure from the first eye of each patient was recorded as the index event and time was measured between the most recent pre-operative gonioscopy date to the index event. Baseline demographics, pre-operative clinical characteristics, glaucoma diagnosis, procedure type, and type of subspecialist performing the procedure were collected.

**Main Outcome Measures:**

Primary outcomes were the percentage of patients who had gonioscopy before a glaucoma procedure (1) at any prior visit and (2) within 1 year prior to the procedure. Secondary outcomes were the baseline factors that were associated with higher gonioscopy utilization at any prior visit using multivariable logistic regression.

**Results:**

The study included 1.1 million patients (mean age 69.5±12.0 years). A majority had an in-office laser surgery (71.9%), while 16.2% had microinvasive glaucoma surgeries (MIGS), 6.5% had a trabeculectomy or tube (traditional), and 4.6% had other glaucoma surgeries. Pre-operative gonioscopy was identified in 64.7% of patients, and 85.0% of those were within 1 year of the index event.

In multivariable models, Asian (OR 1.16, 95%CI 1.13–1.18, *P*<0.001) and Black (OR 1.13, 95%CI 1.12–1.15, *P*<0.001) racial and ethnic groups were associated with higher odds of gonioscopy compared to White groups. When compared to traditional surgery, MIGS were associated with lower utilization (OR 0.69, 95%CI 0.68–0.71, *P*<0.001), but in-clinic laser surgeries were not (*P*=0.231). Glaucoma subspecialists were more likely to perform pre-operative gonioscopy compared to non-glaucoma subspecialists (OR 2.65, 95%CI 2.62–2.69, *P*<0.001).

**Conclusions:**

Pre-operative gonioscopy use and/or coding is lower than expected, given current guidelines. Among glaucoma procedures, ab interno MIGS were associated with lower pre-operative gonioscopy utilization.

## Introduction

Gonioscopy is the gold standard method for assessing angle anatomy during glaucoma evaluations and is also necessary for deciding which glaucoma procedures are feasible for individual patients.^1–3^ It has also been recommended for all patients with glaucoma, open angle suspects, and angle closure suspects according to the American Academy of Ophthalmology Preferred Practice Pattern^®^ guidelines, but is underperformed.^4–6^ Moreover, newer glaucoma procedures increasingly rely on observing angle landmarks and therefore depend on accurate pre-operative gonioscopy.^1,7^ Before the popularization of microinvasive glaucoma surgeries (MIGS), gonioscopy was only performed in about half of Medicare patients in the 5 years leading to glaucoma surgery, which, at the time, was usually a subconjunctival filtering procedure such as a trabeculectomy or tube shunt.^6^

In recent decades, trabeculectomy volumes have decreased while MIGS have grown.^8^ A few reasons for these trends include that MIGS can be combined easily with cataract surgery, have fewer complications, are often approved for mild to moderate glaucoma, and have been recently included in the minimum training requirements for US graduating ophthalmology residents.^7,9^ In recent years, the Laser in Glaucoma and Ocular Hypertension (LiGHT) trial has also shown that selective laser trabeculoplasty (SLT) is an effective primary therapy in patients with open angle glaucoma and ocular hypertension.^10^ Therefore, gonioscopy remains a fundamental skill for all ophthalmologists and will be increasingly important as MIGS and SLT become more widely used.

The American Academy of Ophthalmology IRIS^®^ Registry (Intelligent Research in Sight) has been especially informative for studying practice patterns on a national scale.^11^ As the largest specialty registry, data is not limited to select insurance databases or academic cohorts. Analyzing recommended practices may help us meet the standard of care and improve practice habits. Thus, we sought to analyze nationwide pre-operative gonioscopy utilization patterns for various glaucoma surgeries and laser surgeries using this large registry.

## Methods

A retrospective cohort analysis was conducted from the electronic health record data of patients followed in practices participating in the IRIS Registry. This version of the database was frozen on April 14, 2023.^11^ The IRIS Registry is a centralized data repository and reporting tool that can be used for research purposes. This does not constitute human subject research because data in the IRIS Registry is de-identified, and the investigator does not have access to study identifiers. Therefore, institutional board review and informed consent are not required. This study adheres to the Declaration of Helsinki.

Records of all adult patients (18 years old or over) in the IRIS Registry who underwent glaucoma surgery or laser surgery between January 1, 2014 and April 14, 2023 were included in our analysis. All glaucoma procedures were identified using the Current Procedural Terminology (CPT) codes listed in Supplemental Table 1. Only the first procedure from the first eye (index event) was included in the analysis. If a patient had a bilateral procedure as their first procedure (131,155 patients), one eye was randomly selected for the study using statistical software. We excluded 94,849 patients who had no recorded visits before glaucoma surgery from the same practice. From there, a CPT code search was made for the date of the most recent gonioscopy exam (CPT: 92020) performed before the index event. If gonioscopy laterality was unspecified, it was imputed as bilateral because this is typical practice. The primary outcome was the proportion of patients who had gonioscopy performed before a glaucoma procedure (1) at any prior visit; and (2) within 1 year prior to the procedure. The secondary outcomes studied were the baseline factors that were associated with higher gonioscopy utilization.

Baseline characteristics included age, gender, race and ethnicity, insurance status, geographic region, glaucoma diagnosis, type of glaucoma procedure, ophthalmologist subspecialty, pre-operative visual acuity (VA), and pre-operative intraocular pressure (IOP). The International Classification of Diseases, Ninth and Tenth Revisions (ICD-9/ICD-10) and CPT codes used for glaucoma diagnosis, lens status, and procedure types are listed in Supplemental Table 1. Recorded glaucoma diagnoses were taken from the most recent date prior to the index event (if available) or after the index event only if no diagnoses preceded the index event date. The laterality of the glaucoma procedure and the glaucoma diagnosis had to be congruent, otherwise the diagnosis was classified as “Unknown”. If no glaucoma diagnosis could be identified, then the diagnosis was also “Unknown”. If the ICD code did not indicate laterality, then the glaucoma diagnosis was imputed as bilateral. If a patient had two or more glaucoma diagnoses in the same eye from the same visit, then those patients were grouped together with the “Unspecified Glaucoma” category (Supplemental Table 1).

For this study, a “glaucoma subspecialist” was defined as an ophthalmologist who performed > 25 trabeculectomies and/or tube shunts per year and a “MIGS subspecialist” was someone who performed > 50 MIGS per year but did not meet the definition of a glaucoma subspecialist (the two designations are hierarchically exclusive of each other).^8^ For lens status, patients were classified as being phakic if they did not have an aphakia or pseudophakia ICD code or a cataract surgery CPT code before the index event, pseudophakic if they had a pseudophakia ICD code and/or had a cataract surgery CPT code (without an aphakia ICD code) before the index event, or aphakic if they had an aphakia ICD code before the index event. Lastly, pre-operative VA and IOP were estimated as the mean of recorded measurements over the 12 months prior to the index event to obtain more stable estimates for each patient and to reduce the influence of measurement error.^12^

### Statistical Analysis

Descriptive statistics were used to report baseline characteristics of patients receiving gonioscopy versus no gonioscopy before glaucoma procedures. For time between gonioscopy to procedure, we limited our analysis to patients who received gonioscopy and presented density plots by glaucoma procedure type. For factors associated with pre-operative gonioscopy, we used multivariable logistic regression to estimate the probability of receiving gonioscopy as a function of demographic (e.g., age, sex, etc.) and clinical variables (e.g., pre-operative IOP, VA, etc.). We used an alpha level (*P*-value threshold) of 0.05 for significance testing and used the R statistical programming language (version 4.4.1) to conduct the analysis.

## Results

In total, 1,082,136 adult patients had at least one glaucoma surgery or laser surgery in the IRIS Registry during the study period (Table 1). The average age of patients was 69.5 ± 12.0 years (57.6% female) at the time of their procedure. The majority received an in-clinic laser surgery (71.9%), while 16.2% had a MIGS, 6.5% had a traditional glaucoma surgery, 4.6% had other glaucoma procedures, and < 1% had combined glaucoma procedures on the same day. The most common diagnosis was primary open angle glaucoma (POAG, 52.6%), followed by unspecified glaucoma (15.6%), primary angle closure suspects (PACS, 12.4%), open angle glaucoma suspects (9.7%), primary angle closure glaucoma (PACG, 3.4%), pseudoexfoliation glaucoma (1.7%), unknown diagnosis/no recorded diagnosis (1.6%), other glaucoma (1.5%), pigmentary glaucoma (0.9%), uveitic glaucoma (0.4%), and traumatic glaucoma (0.3%). Over 63% of patients were identified as White race, while 13.4% and 3.7% were identified as Black or Asian race, respectively (17.2% were unknown). About 9.8% of patients were of Hispanic ethnicity (22.7% were unknown). Commercial (20.4%) and Medicare (23.0%) were the two most common insurance types (40.1% were unknown). Lastly, 76.6% of patients were phakic in the procedural eye at the time of the index event.

**Figure 1:**
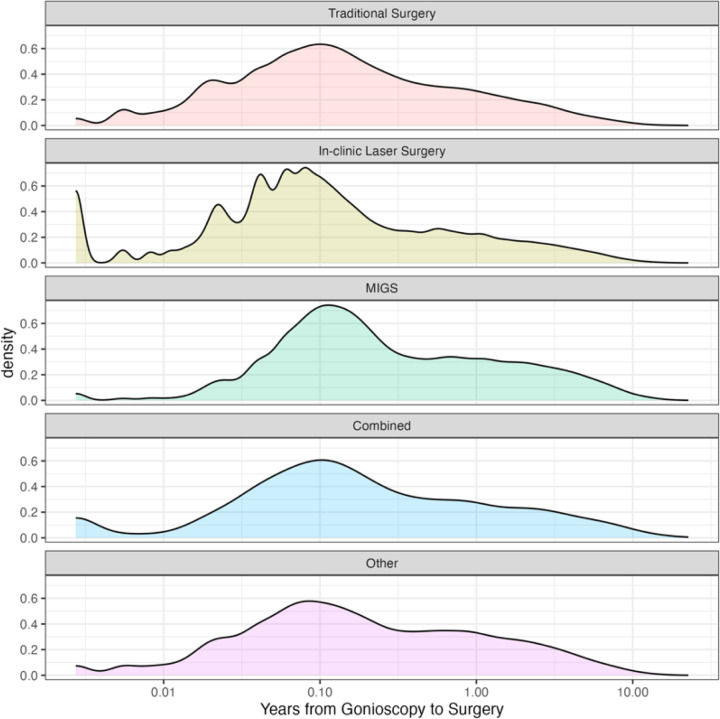
Patient Distribution Of Time From Gonioscopy To Procedure (Log Scale) By Each Procedure Type.

Pre-operative gonioscopy was performed in 64.7% of patients in the IRIS Registry. Of those who had pre-operative gonioscopy, 85.0% of patients had their gonioscopy performed within 1 year of the index event (Fig. 1). Pre-operative gonioscopy was identified in 69.7% of patients undergoing traditional glaucoma surgery, 67.1% before in-clinic laser surgery, 55.5% before an ab interno MIGS, 51.2% before combined glaucoma procedures, and 54.2% before other types of glaucoma procedures (*P* < 0.001). When sorting by subspecialist performing the glaucoma procedure, 78.3% had pre-operative gonioscopy when done by glaucoma subspecialists, 62.3% by MIGS subspecialists, and 60.1% by non-subspecialists (*P* < 0.001). By lens status, 67.0% of phakic patients had pre-operative gonioscopy, while only 57.5% of pseudophakic patients and 52.9% of aphakic patients received it (*P* < 0.001). Lastly, pre-operative gonioscopy utilization was identified in 68.1% of patients with commercial insurance, 63.6% with Medicare, and 58.2% of uninsured patients (*P* < 0.001).

**Figure 2:**
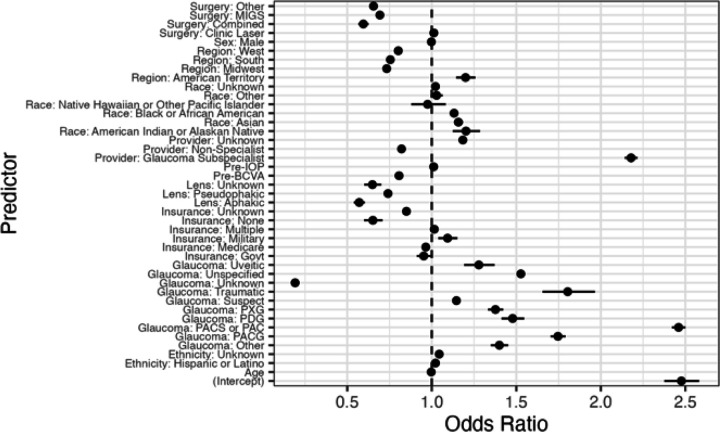
Forest Plot of Predictors of Gonioscopy Utilization Before Glaucoma Procedures. Notes: MIGS=microinvasive glaucoma surgery; Pre-IOP=pre-operative intraocular pressure; pre-BCVA=pre-operative best corrected visual acuity; Govt=Government; PXG=pseudoexfoliation glaucoma; PDG=pigmentary glaucoma; PACG=primary angle closure glaucoma; PACS=Primary angle closure suspect; PAC=Primary angle closure

In a multivariable logistic regression analysis, higher gonioscopy utilization at any time prior to the index event was associated with American Indian or Alaska Native, Asian, Black, and Hispanic racial and ethnic groups, phakic status, primary angle closure spectrum diagnoses, secondary open angle glaucoma diagnoses, and glaucoma subspecialists performing the procedure (Table 2 and Fig. 2). When compared to White race, American Indian or Alaska Native (1.20, 95% CI 1.12–1.29, *P* < 0.001), Asian (OR 1.16, 95% CI 1.13–1.18, *P* < 0.001), and Black (OR 1.13, 95% CI 1.12–1.15, *P* < 0.001) racial groups were associated with higher odds of receiving pre-operative gonioscopy. Hispanic ethnicity (OR 1.02, 95% CI 1.00–1.04, *P* = 0.012) was associated with higher odds of pre-operative gonioscopy than non-Hispanic ethnicity. When compared to POAG, PACS (OR 2.46, 95% CI 2.42–2.50, *P* < 0.001), PACG (OR 1.75, 95% CI 1.70–1.79, *P* < 0.001), and open angle glaucoma suspects (OR 1.15, 95% CI 1.13–1.16, *P* < 0.001) had higher odds of pre-operative gonioscopy. Lastly, glaucoma subspecialists (OR 2.18, 95% CI 2.14–2.22, *P* < 0.001) were associated with higher odds and non-subspecialists (OR 0.82, 95% CI 0.81–0.83, *P* < 0.001) were associated with lower odds of having had pre-operative gonioscopy when compared to if a MIGS subspecialist was performing the procedure.

Lower pre-operative gonioscopy utilization was associated with MIGS, combined glaucoma procedures, uninsured status, patients with aphakia and pseudophakia, worse pre-operative VA, and non-subspecialists performing the glaucoma procedure (Table 2 and Fig. 2). When compared to traditional glaucoma surgery, MIGS (OR 0.69, 95% CI 0.68–0.71, *P* < 0.001) and combined glaucoma procedures (OR 0.60, 95% CI 0.57– 0.63, *P* < 0.001) had lower likelihoods of having pre-operative gonioscopy, but in-clinic laser surgeries did not (*P* = 0.231). Pseudophakic (OR 0.74, 95% CI 0.73–0.75, *P* < 0.001) and aphakic patients (OR 0.57, 95% CI 0.54–0.60, *P* < 0.001) were associated with lower utilization than phakic patients. Uninsured patients were associated with lower utilization than patients with commercial insurance (OR 0.65, 95% CI 0.60–0.71, *P* < 0.001). Lastly, an unknown glaucoma diagnosis/no recorded diagnosis (OR 0.19, 95% CI 0.18–0.20, *P* < 0.001) was also associated with lower odds of having pre-operative gonioscopy.

## Discussion

In the IRIS Registry, pre-operative gonioscopy usage was lower than expected among every glaucoma surgery and laser surgery category based on the American Academy of Ophthalmology Preferred Practice Pattern guidelines.^1^ However, most pre-operative gonioscopy when performed was done within 1 year of the procedure. Higher pre-operative gonioscopy utilization was associated with American Indian or Alaska Native, Asian, Black, and Hispanic racial and ethnic groups, phakic status, primary angle closure spectrum diagnoses, secondary open angle glaucoma diagnoses, and glaucoma subspecialists performing the glaucoma procedure. In contrast, lower utilization was associated with MIGS, combined glaucoma procedures, uninsured status, patients with aphakia and pseudophakia, worse pre-operative VA, and non-subspecialists performing the glaucoma procedure. While the Academy has recommended gonioscopy evaluation for diagnosis of patients with glaucoma in their Preferred Practice Pattern guidelines, they also recommended pre-operative gonioscopy for proper patient selection, especially for MIGS.^1^

Despite its versatility and low technological requirements, routine gonioscopy has remained an underperformed skill. Between 1997 to 1999, 46% of patients with POAG had gonioscopy recorded on the initial visit in a study using administrative and chart data from six healthcare plans.^4^ In a recent study of the Optum Clinformatics Data Mart, only 30% of patients evaluated for glaucoma between 2009–2020 had gonioscopy recorded within the first six months of the initial visit.^13^ The authors also found that White race and pseudophakic patients were associated with lower recorded gonioscopy utilization, which is consistent with our study results. This may be because White race and pseudophakia have both been associated with lower primary angle closure spectrum diagnoses.^2^ However, gonioscopy should not be skipped because many secondary open angle glaucomas, including pigmentary, pseudoexfoliation, and angle recession glaucomas, still need gonioscopy for diagnosis. Low pre-operative gonioscopy utilization is not a new problem either. Coleman and colleagues found that 49% of Medicare patients had pre-operative gonioscopy within the 5 years leading up to their glaucoma procedure between 1995 to 1999.^6^ However, that study was published before the first MIGS implant was introduced in 2012, and MIGS volumes have risen since then.^8^

We found that ab interno MIGS procedures were associated with lower gonioscopy utilization than traditional glaucoma surgery and in-clinic laser surgeries. In one study by Rathi and colleagues, they found that a majority of iStent surgeries and almost half of goniotomies were performed by non-subspecialists in 2016 using a Medicare claims database.^8^ In our study, we found that non-subspecialists performing the glaucoma procedure was an independent risk factor for lower pre-operative gonioscopy utilization. Greater awareness for pre-operative gonioscopy during MIGS training may help with this habit as more non-subspecialists are adopting MIGS in their practice and because many non-subspecialists may have finished ophthalmology residency several years ago. Also, while in-clinic laser surgeries were not associated with lower utilization in our study, SLT has been growing in interest as a preferred first-line glaucoma treatment among both glaucoma subspecialists and non-subspecialists in a recent survey by the American Society of Cataract and Refractive Surgery.^14^

Primary angle closure spectrum diagnoses including suspects were associated with higher pre-operative gonioscopy utilization than patients with POAG. Despite PACG being less common than POAG, PACG contributes toward a much higher risk of blindness worldwide.^15^ Several studies have also shown the higher prevalence of angle closure in Asian and Native Alaskan populations.^16–18^ Therefore, it is understandable that these study groups and phakic status were associated with higher pre-operative gonioscopy use in our study. In the study by Coleman and colleagues, they also found that higher gonioscopy utilization was associated with primary angle closure spectrum diagnoses and patients undergoing laser iridotomy.^6^

Black race, Hispanic ethnicity, and uninsured status have been historically associated with healthcare disparities. Although Black race has been associated with POAG and POAG-related blindness, angle closure can still occur in the Black population.^19–21^ In one study by Thompson and colleagues, Black race was also associated with greater missed diagnosis of angle closure by Van Herick technique, which was confirmed by subsequent gonioscopy.^20^ Furthermore, Black race and Hispanic ethnicity have both been associated with higher odds of PACG-related blindness in the IRIS Registry.^21^ In our study, we found that Black race and Hispanic ethnicity were associated with higher pre-operative gonioscopy utilization, which is critical for proper diagnosis and surgical planning in this vulnerable population. Previous studies have not considered gonioscopy utilization among the uninsured population. Uninsured patients would have less access to routine ophthalmologic examinations, which could therefore lead to missed, incorrect, or late glaucoma diagnoses prior to needing surgery.

There are several limitations to this large retrospective registry-based investigation. Gonioscopy usage was identified using CPT coding in the IRIS Registry and providers may have been undercoding this examination technique.^6^ However, our assumption is that if this were the case, undercoding would be similar across different glaucoma surgery and laser surgery categories. Undercoding may be different across subspecialties, which could explain why we found that glaucoma subspecialists had higher pre-operative utilization. Non-subspecialists may not know that gonioscopy is a billable code. There could also be miscoding errors for glaucoma diagnoses when using ICD codes from a large registry. Gonioscopy may have been performed prior to when practices began sharing data with the IRIS Registry. This could explain why we found that having an unknown glaucoma diagnosis was associated with low pre-operative gonioscopy. To mitigate this, our study included patients who had a glaucoma procedure starting on January 1, 2014, rather than the year prior which is when the IRIS Registry began collecting data. Only < 2% of patients had an unknown glaucoma diagnosis using this method. We also did not directly have data on ophthalmologists’ fellowship background and therefore used an indirect method of determining whether one was a glaucoma subspecialist based on the number of trabeculectomies or tube shunts one had performed. This method has been used in other large registry studies previously.^8^ The IRIS Registry also does not have social determinants of health information besides insurance status. Lastly, the IRIS Registry contains data from mostly community-based practices so our results may not be generalizable to other practices that do not contribute to the IRIS Registry.

In conclusion, gonioscopy usage before glaucoma procedures was lower than recommended as shown by the IRIS Registry. While in-office glaucoma laser surgeries and ab interno MIGS both rely on observing angle landmarks during the procedure, MIGS and non-subspecialists performing glaucoma procedures were each associated with lower utilization. Uninsured patients were another group at risk of not having had gonioscopy before glaucoma procedures. Future studies should explore how the utilization patterns of other angle imaging modalities contribute to these observations, and whether these different practice patterns result in different surgical outcomes. Local efforts to improve pre-operative gonioscopy habits, including during MIGS training, may help improve future practice patterns.

## Supplementary Material

Supplement 1

## Figures and Tables

**Table 1:** Patterns of Gonioscopy Utilization Before Glaucoma Procedures

	No Gonioscopy^1^ 381,862 (35%)	Had Gonioscopy^1^ 700,274 (65%)	P-value^2^
Gonioscopy within 1 year before procedure	0	595,232 (85%)	< 0.001
Age at procedure (years)	72 (64, 79)	70 (62, 77)	< 0.001
Sex			< 0.001
Female	216,404 (35%)	406,568 (65%)	
Male	165,458 (36%)	293,706 (64%)	
Race			< 0.001
American Indian or Alaska Native	1,345 (30%)	3,169 (70%)	
Asian	12,499 (31%)	27,512 (69%)	
Black	48,676 (33%)	96,738 (67%)	
Native Hawaiian or Other Pacific Islander	641 (36%)	1,159 (64%)	
Other	6,499 (33%)	13,440 (67%)	
Unknown	62,543 (34%)	123,262 (66%)	
White	249,659 (36%)	434,994 (64%)	
Ethnicity			< 0.001
Hispanic or Latino	34,505 (33%)	71,630 (67%)	
Not Hispanic or Latino	263,944 (36%)	466,518 (64%)	
Unknown	83,413 (34%)	162,126 (66%)	
Insurance Status			< 0.001
Commercial	70,231 (32%)	150,104 (68%)	
Government	3,622 (35%)	6,845 (65%)	
Medicare	90,679 (36%)	158,430 (64%)	
Military	2,445 (33%)	4,987 (67%)	
Multiple	55,132 (35%)	103,223 (65%)	
No insurance	1,070 (42%)	1,491 (58%)	
Unknown	158,683 (37%)	275,194 (63%)	
Lens Status			< 0.001
Aphakic	2,541 (47%)	2,857 (53%)	
Phakic	274,053 (33%)	555,316 (67%)	
Pseudophakic	103,897 (43%)	140,552 (57%)	
Unknown	1,371 (47%)	1,549 (53%)	
Pre-operative VA	0.18 (0.07, 0.36)	0.14 (0.05, 0.30)	< 0.001
Pre-operative IOP	17.5 (15.0, 20.8)	17.7 (15.0, 21.0)	< 0.001
Procedure Type			< 0.001
Ab interno MIGS	78,074 (44%)	97,555 (56%)	
Combined	3,641 (49%)	3,824 (51%)	
In-clinic laser surgery	255,840 (33%)	522,576 (67%)	
Other	22,884 (46%)	27,132 (54%)	
Traditional	21,423 (30%)	49,187 (70%)	
Provider Type			< 0.001
Glaucoma Subspecialist	40,385 (22%)	145,681 (78%)	
MIGS Subspecialist	42,143 (38%)	69,513 (62%)	
Non-Subspecialist	241,259 (40%)	363,558 (60%)	
Unknown	58,075 (32%)	121,522 (68%)	
Glaucoma Type			< 0.001
Open Angle Suspect	36,805 (35%)	67,765 (65%)	
Other Glaucoma	5,301 (33%)	10,545 (67%)	
Pigmentary Glaucoma	2,844 (29%)	7,000 (71%)	
POAG or NTG	231,381 (41%)	337,706 (59%)	
Primary Angle Closure Glaucoma	9,346 (25%)	27,581 (75%)	
Primary Angle Closure Suspect/Primary Angle Closure	24,775 (19%)	109,126 (81%)	
Pseudoexfoliation Glaucoma	5,907 (32%)	12,486 (68%)	
Traumatic Glaucoma	788 (27%)	2,138 (73%)	
Unknown	13,290 (77%)	4,007 (23%)	
Unspecified Glaucoma	50,163 (30%)	119,183 (70%)	
Uveitic Glaucoma	1,262 (32%)	2,737 (68%)	
Region			< 0.001
Midwest	84,629 (39%)	132,428 (61%)	
Northeast	67,439 (29%)	163,274 (71%)	
South	150,532 (36%)	263,056 (64%)	
Unknown	3593 (41%)	5137 (59%)	
US Territories	2,610 (25%)	7,996 (75%)	
West	73,059 (36%)	128,383 (64%)	

VA = visual acuity; IOP = intraocular pressure; MIGS = microinvasive glaucoma surgery; POAG = primary open angle glaucoma, NTG = normal tension glaucoma

1 n (%); Median (Q1, Q3)

2 Pearson’s Chi-squared test; Wilcoxon rank sum test

**Table 2:** Multivariable Logistic Regression Analysis of Gonioscopy Utilization Before Glaucoma Procedures

	Odds Ratios (95% CI)	P-value	
Age at procedure (years)	1.00 (1.00–1.00)	< 0.001	
Male	1.00 (0.99–1.01)	0.562	
Race			
American Indian or Alaska Native	1.20 (1.12–1.29)		< 0.001
Asian	1.16 (1.13–1.18)		< 0.001
Black	1.13 (1.12–1.15)		< 0.001
Native Hawaiian or Other Pacific Islander	0.98 (0.88–1.08)		0.634
Other	1.03 (0.99–1.06)		0.116
Unknown	1.02 (1.01–1.04)		0.002
White	1.00		
Ethnicity			
Hispanic or Latino	1.02 (1.00–1.04)		0.012
Not Hispanic or Latino	1.00		
Unknown	1.04 (1.03–1.06)		< 0.001
Insurance Status			
Commercial	1.00		
Government	0.95 (0.91–0.99)		0.026
Medicare	0.96 (0.95–0.98)		< 0.001
Military	1.09 (1.04–1.15)		0.001
Multiple	1.01 (1.00–1.03)		0.050
No insurance	0.65 (0.60–0.71)		< 0.001
Unknown	0.85 (0.84–0.86)		< 0.001
Lens Status			
Aphakic	0.57 (0.54–0.60)		< 0.001
Phakic	1.00		
Pseudophakic	0.74 (0.73–0.75)		< 0.001
Unknown	0.65 (0.60–0.70)		< 0.001
Pre-operative VA	0.81 (0.80–0.81)		< 0.001
Pre-operative IOP	1.01 (1.01–1.01)		< 0.001
Procedure Type			
Ab interno MIGS	0.69 (0.68–0.71)		< 0.001
Combined	0.60 (0.57–0.63)		< 0.001
In-clinic laser surgery	1.01 (0.99–1.03)		0.231
Other	0.66 (0.64–0.67)		< 0.001
Traditional	1.00		
Provider Type			
Glaucoma Subspecialist	2.18 (2.14–2.22)		< 0.001
MIGS Subspecialist	1.00		
Non-Subspecialist	0.82 (0.81–0.83)		< 0.001
Unknown	1.18 (1.16–1.20)		< 0.001
Glaucoma Type			
Open Angle Suspect	1.15 (1.13–1.16)		< 0.001
Other Glaucoma	1.40 (1.35–1.45)		< 0.001
Pigmentary Glaucoma	1.48 (1.41–1.55)		< 0.001
POAG or NTG	1.00		
Primary Angle Closure Glaucoma	1.75 (1.70–1.79)		< 0.001
Primary Angle Closure Suspect/Primary Angle Closure	2.46 (2.42–2.50)		< 0.001
Pseudoexfoliation Glaucoma	1.38 (1.33–1.42)		< 0.001
Traumatic Glaucoma	1.80 (1.66–1.97)		< 0.001
Unknown	0.19 (0.18–0.20)		< 0.001
Unspecified Glaucoma	1.53 (1.51–1.55)		< 0.001
Uveitic Glaucoma	1.28 (1.19–1.37)		< 0.001
Region			
Midwest	0.73 (0.72–0.74)		< 0.001
Northeast	1.00		
South	0.75 (0.75–0.76)		< 0.001
US Territories	1.20 (1.14–1.26)		< 0.001
West	0.80 (0.79–0.81)		< 0.001
(Intercept)	2.48 (2.38–2.58)		< 0.001

CI = confidence interval; VA = visual acuity; IOP = intraocular pressure; MIGS = microinvasive glaucoma surgery; POAG = primary open angle glaucoma, NTG = normal tension glaucoma

## References

[1] Gedde SJ, Vinod K, Wright MM, Primary Open-Angle Glaucoma Preferred Practice Pattern^®^. Ophthalmology. 2021;128(1):P71–P150. 34933745 10.1016/j.ophtha.2020.10.022

[2] Gedde SJ, Chen PP, Muir KW, Primary Angle-Closure Disease Preferred Practice Pattern^®^. Ophthalmology. 2021;128(1):P30–P70. 34933744 10.1016/j.ophtha.2020.10.021

[3] Gedde SJ, Lind JT, Wright MM, Primary Open-Angle Glaucoma Suspect Preferred Practice Pattern^®^. Ophthalmology. 2021;128(1):P151–P192. 34933743 10.1016/j.ophtha.2020.10.023

[4] Fremont AM, Lee PP, Mangione CM, Patterns of Care for Open-Angle Glaucoma in Managed Care. Arch Ophthalmol. 2003;121(6):777–783. 12796247 10.1001/archopht.121.6.777

[5] Stanley J, Huisingh CE, Swain TA, Compliance With Primary Open-angle Glaucoma and Primary Open-angle Glaucoma Suspect Preferred Practice Patterns in a Retail-based Eye Clinic. J Glaucoma. 2018;27(12):1068–1072. 30234750 10.1097/IJG.0000000000001093 PMC6265080

[6] Coleman AL, Yu F, Evans SJ. Use of Gonioscopy in Medicare Beneficiaries Before Glaucoma Surgery. J Glaucoma. 2006;15(6):486–493. 17106360 10.1097/01.ijg.0000212287.62798.8f

[7] Samuelson TW, Sarkisian SR, Lubeck DM, Prospective, Randomized, Controlled Pivotal Trial of an Ab Interno Implanted Trabecular Micro-Bypass in Primary Open-Angle Glaucoma and Cataract: Two-Year Results. Ophthalmology. 2019;126(6):811–821. 30880108 10.1016/j.ophtha.2019.03.006

[8] Rathi S, Andrews CA, Greenfield DS, Stein JD. Trends in Glaucoma Surgeries Performed by Glaucoma Subspecialists versus Nonsubspecialists on Medicare Beneficiaries from 2008 through 2016. Ophthalmology. 2021;128(1):30–38. 32598949 10.1016/j.ophtha.2020.06.051 PMC7755669

[9] Ahmed IIK, De Francesco T, Rhee D, Long-term Outcomes from the HORIZON Randomized Trial for a Schlemm’s Canal Microstent in Combination Cataract and Glaucoma Surgery. Ophthalmology. 2022;129(7):742–751. 35218867 10.1016/j.ophtha.2022.02.021

[10] Gazzard G, Konstantakopoulou E, Garway-Heath D, Selective laser trabeculoplasty versus eye drops for first-line treatment of ocular hypertension and glaucoma (LiGHT): a multicentre randomised controlled trial. Lancet. 2019;393(10180):1505–1516. 30862377 10.1016/S0140-6736(18)32213-X PMC6495367

[11] Chiang MF, Sommer A, Rich WL, Lum F, Parke DW 2nd. The 2016 American Academy of Ophthalmology IRIS^®^ Registry (Intelligent Research in Sight) Database: Characteristics and Methods. Ophthalmology. 2018;125(8):1143–1148. 29342435 10.1016/j.ophtha.2017.12.001

[12] Brant A, Kolomeyer N, Goldberg JL, Evaluating Visual Acuity in the American Academy of Ophthalmology IRIS^®^ Registry. Ophthalmol Sci. 2023 Jun 19;4(1):100352. 37869025 10.1016/j.xops.2023.100352 PMC10587626

[13] Lee JH, Yoo K, Lung K, Patterns and Disparities in Recorded Gonioscopy During Initial Glaucoma Evaluations in the United States. Am J Ophthalmol. 2024;264:90–98. 38423202 10.1016/j.ajo.2024.02.032 PMC11257810

[14] Rhee DJ, Sancheti H, Rothman AL, Primary Practice Patterns for the Initial Management of Open Angle Glaucoma. J Glaucoma. 2024;33(9):671–678. 38874528 10.1097/IJG.0000000000002453

[15] Quigley HA, Broman AT. The number of people with glaucoma worldwide in 2010 and 2020. Br J Ophthalmol. 2006;90(3):262–7. 16488940 10.1136/bjo.2005.081224 PMC1856963

[16] Seah SK, Foster PJ, Chew PT, Incidence of acute primary angle-closure glaucoma in Singapore. An island-wide survey. Arch Ophthalmol. 1997;115(11):1436–40. 9366676 10.1001/archopht.1997.01100160606014

[17] Nguyen N, Mora JS, Gaffney MM, A high prevalence of occludable angles in a Vietnamese population. Ophthalmology. 1996;103(9):1426–31. 8841301 10.1016/s0161-6420(96)30488-0

[18] Arkell SM, Lightman DA, Sommer A, The prevalence of glaucoma among Eskimos of northwest Alaska. Arch Ophthalmol. 1987;105(4):482–5. 3566600 10.1001/archopht.1987.01060040052031

[19] Friedman DS, Jampel HD, Muñoz B, West SK. The prevalence of open-angle glaucoma among blacks and whites 73 years and older: the Salisbury Eye Evaluation Glaucoma Study. Arch Ophthalmol. 2006;124(11):1625–30. 17102012 10.1001/archopht.124.11.1625

[20] Thompson AC, Vu DM, Cowan LA, Asrani S. Risk Factors Associated with Missed Diagnoses of Narrow Angles by the Van Herick Technique. Ophthalmol Glaucoma. 2018;1(2):108–114. 32672561 10.1016/j.ogla.2018.08.002

[21] Shah SN, Zhou S, Sanvicente C, Prevalence and Risk Factors of Blindness Among Primary Angle Closure Glaucoma Patients in the United States: An IRIS Registry Analysis. Am J Ophthalmol. 2024;259:131–140. 37944688 10.1016/j.ajo.2023.11.007 PMC10922147

