# Supplementary material for: Factors Associated with Gonioscopy Before Glaucoma Procedures in the IRIS® Registry
Source: Res Sq. 2025 Jan 13:rs.3.rs-5789587. Preprint. [Version 1] doi: 10.21203/rs.3.rs-5789587/v1 (PMC11774446; doi:10.21203/rs.3.rs-5789587/v1)
Supplement: Supplement 1 [file NIHPPRS5789587v1-supplement-1.pdf]

Supplementary Files

This is a list of supplementary files associated with this preprint. Click to download.

- SupplementoryTable.docx
